# Efficacy of Chinese herbal injections combined with fluoropyrimidine and oxaliplatin-based chemotherapy for advanced colorectal cancer: A protocol for systematic review and meta-analysis of randomized controlled trials

**DOI:** 10.1097/MD.0000000000023550

**Published:** 2020-12-24

**Authors:** Shuo Wang, Xueqian Wang, Ying Zhang, Tong Zhou, Shuaihang Hu, Peiyu Tian, Zheng Li, Yuxiao Li, Yuerong Gui, Jun Dong, Wei Hou

**Affiliations:** a Guang’an men Hospital, China Academy of Chinese Medical Sciences; b Beijing University of Chinese Medicine, China.

**Keywords:** advanced colorectal cancer, Chinese herbal injections, efficacy, randomized controlled trial, systematic review

## Abstract

**Background:**

Fluoropyrimidine combined with oxaliplatin-based chemotherapy have become the first-line treatment for advanced colorectal cancer (CRC). Chinese herbal injections (CHIs), as an important part of TCM, have been widely applied as adjunctive treatments to chemotherapy in patients with advanced CRC. However, the efficacy of this combination therapy has not been evaluated comprehensively.

**Methods:**

We will conduct this systematic review and meta-analysis in accordance with the Preferred Reported Items for Systematic Review and Meta-analysis (PRISMA) guidelines. 7 databases will be searched for related randomized controlled trials (RCTs) from their inception to August 31, 2020: PubMed, Cochrane Library, EMBASE, China National Knowledge Infrastructure (CNKI), VIP Database for Chinese Technical Periodicals (VIP), SinoMED and Wanfang Database. Two researchers will perform study selection, data extraction, and assessment of risk of bias independently. The primary outcomes are the disease control rate (DCR) and the objective response rate (ORR), the secondary outcomes are progression-free survival (PFS), survival rate, quality of life (QoL) and adverse effects. Cochrane Review Manager (RevMan 5.3) software will be used to analyze the outcomes.

**Results and Conclusion:**

This systematic review will evaluate the efficacy of CHIs and fluoropyrimidine and oxaliplatin-based chemotherapy for advanced CRC so as to provide valuable evidence to the application of CHIs in advanced CRC.

**Systematic review registration:**

INPLASY registration number: INPLASY2020100050.

## 1 Introduction

According to Global Cancer Statistics 2018, over 1.8 million new colorectal cancer (CRC) cases and 881,000 deaths are estimated to occur in 2018, accounting for about 1 in 10 cancer cases and deaths. Overall, CRC ranks third in terms of incidence but second in terms of mortality.^[1]^ In America, the latest statistical data of the National Cancer Institute (NIH) SEER database shows that the estimated new CRC cases in 2020 have reached 147,950, counts 8.2% of all new cancer cases. The estimated CRC deaths in 2020 have reached 53,200, counts 8.8% of all cancer deaths, leading a serious threat to peoples health and life.^[2]^

Surgery is the cornerstone of CRC therapy. Unfortunately, 30% to 40% of patients with CRC are confirmed in its advanced stage and lose the chance of surgery,^[3]^ so chemotherapy regimen has been the optimal treatment.^[4]^ Fluoropyrimidines (FUs), including 5-fluorouracil (5-FU) and capecitabine,^[5]^ blocking DNA and RNA synthesis by inhibiting thymidylate synthase,^[6]^ has widely prescribed for CRC. FUs and oxaliplatin-based chemotherapies, such as FOLFOX (5-FU plus oxaliplatin plus leucovorin), CAPEOX or XELOX (capecitabine plus oxaliplatin) and FOLFOXIRI (irinotecan plus 5-FU plus oxaliplatin plus leucovorin), are key components of the first-line treatment for advanced CRC. Although the addition of targeted drugs like bevacizumab and cetuximab enables prolonged median survival time from less than 1 year when treated 5-FU alone to more than 2 years now,^[7]^ fluoropyrimidine and oxaliplatin-based chemotherapy is still the cornerstone. However, it still leave much to be desired, the short- and long-term efficacy of chemotherapies needs to be improved, and patients undergoing chemotherapy may suffer from many side effects, such as bone marrow suppression, nausea, vomiting, etc., which have become major impediment in the treatments of advanced CRC and lead a reduced quality of life.

Traditional Chinese medicine (TCM) is one of the most important treatment methods in advanced CRC therapies, and has been widely used in China and other parts of Asia. Chinese herbal injections (CHIs), prepared by extracting and purifying effective ingredients from Chinese herbal medicines,^[8]^ are an important part of TCM. CHIs have simpler components, quicker onset of action, clearer indication, and no intestinal absorption compared with classic Chinese herbal medicine administration methods.^[9–10]^ Many researches indicated that it had obvious advantages in reducing toxicity of chemotherapy, enhacing life quality and improving short- and long-term efficacy. For example, Shenqi Fuzheng Injection (SFI) has been reported to have effects in improving the efficacy of chemotherapy in patients with advanced CRC and reducing myelosuppression and gastrointestinal adverse effects caused by chemotherapy.^[11]^ Kushen injection has been demonstrated to comprehensively reducing the incidence of nausea and vomitting, improving chemotherapy tolerance and quality of life, and helping promote the objective response rate (ORR) of patients with advanced CRC who undergoing FOLFOX4 chemotherapy.^[12]^

In China, CHIs have been widely used in advanced CRC patients, and a large amount of medical literatures have been published, it can be seen that CHIs play an important complementary role in enhancing efficacy and reducing toxicity during chemotherapy of advanced CRC. However, a systematic review has not been found. We will carry out this study to give more clinical evidence of CHIs in patients with advanced CRC who accepted fluoropyrimidine and oxaliplatin-based chemotherapy so as to guide clinical practice in the future.

## 2 Method

### 2.1 Study registration

This systematic review has been registered on INPLASY as INPLASY2020100050 (https://inplasy.com/inplasy-2020-10-0050/) and will be developed according to the Preferred Reporting Items for Systematic Reviews and Meta-Analysis Protocol (PRISMA-P) statement guidelines. This study does not require ethical approval as all the research materials are published studies.

### 2.2 Data sources and search strategy

A comprehensive search will be conducted from inception to August 31, 2020 in 7 electronic medical databases, including 3 English-language databases (PubMed, EMBASE, Cochrane) and 4 Chinese-language databases (China National Knowledge Infrastructure [CNKI], Wanfang Data, VIP Database for Chinese Technical Periodicals [VIP], SinoMed). The search strategy in PubMed was showed on Table 1.

**Table 1:** Search strategy for PubMed.

Number	Search terms
#1	(Colorectal Neoplasms[MeSH] OR Colonic Neoplasms[MeSH]) OR Rectal Neoplasms[MeSH]
#2	(tumor^∗^[tiab] OR carcinoma^∗^[tiab] OR neoplasm^∗^[tiab] OR cancer^∗^[tiab]) AND (coloretal[tiab] OR colon[tiab] OR colonic[tiab] OR rectal[tiab] OR retum[tiab])
#3	#1 OR #2
#4	Chinese herbal injection^∗^[tiab] OR Chinese medicine injection[tiab] injection of TCM[tiab] OR Shenqifuzheng[tiab] OR Kanglaite[tiab] OR Compound Kushen[tiab] OR Fufangkushen[tiab] OR Compound matrine[tiab] OR Aidi[tiab] OR Cinobufotalin injection[tiab] OR Huachansu[tiab] OR Xiaoaiping[tiab] OR Xiao-Ai-Ping[tiab] OR Marsdenia Tenacissima[tiab] OR Elemene[tiab] OR Lanxiangxi[tiab] OR Xiangguduotang[tiab] OR lentinan[tiab] OR javanica oil emulsion[tiab] OR Bmcea javanica[tiab] OR Yadanziyouru[tiab] OR kang’ai[tiab] OR kangai[tiab] OR kang-ai[tiab] OR Huangqi[tiab] OR Astragalus[tiab] OR Shenfu[tiab] OR Shenmai[tiab]
#5	#3 AND #4

### 2.3 Inclusion criteria

#### 2.3.1 Types of studies

Only randomized controlled trials (RCTs) will be selected and assessed for inclusion.

#### 2.3.2 Types of participants

Patients with advanced CRC should meet the following criteria:

Inclusion criteria:

1. Patients included in each trails were cytologically or pathologically confirmed cases of CRC.
2. Patients belonging to Stage III or IV according to American Joint Committee on Cancer Staging System (8th edition).
3. Participants in treatment group received CHIs combined with fluoropyrimidine (5-FU or capecitabine) and oxaliplatin-based chemotherapy, with or without bevacizumab or cetuximab.
4. The control group was only given fluoropyrimidine (5-FU or capecitabine) and oxaliplatin-based chemotherapy, with or without bevacizumab or cetuximab.
5. CHIs are given intravenously.
6. Reported at least one of the outcomes of interest.

Exclusion criteria:

1. Duplicated publications or overlapping study population.
2. Multiple TCM intervention in the treatment group.
3. Documents of data errors.
4. Off-label use of CHIs.

#### 2.3.3 Types of interventions

The intervention in the treatment group is CHIs combined with fluoropyrimidine (5-FU or capecitabine) and oxaliplatin-based chemotherapy, with or without bevacizumab or cetuximab. The intervention in the control group is fluoropyrimidine (5-FU or capecitabine) and oxaliplatin-based chemotherapy, with or without bevacizumab or cetuximab.

#### 2.3.4 Types of outcome measures

Primary outcomes:

1. Objective response rate (ORR): According to WHO^[13]^ guidelines for solid tumor responses or Response Evaluation Criteria in Solid Tumors (RECIST),^[14]^ the tuomr responses were evaluated as complete response (CR), partial response (PR), stable disease (SD), and progressive disease (PD). ORR refers to the proportion of patients with CR plus PR.
2. Disease control rate (DCR), calculated as the proportion of patients with CR plus PR plus SD.

Secondary outcomes:

1. Progression-free survival (PFS), the time from study entry to relapse or death.^[15]^
2. Survival rate, the proportion of participants alive at the beginning of a time interval who survive to the end of the interval.
3. Quality of life (QoL), evaluated mainly by scales, such as Karnofsky performance scale (KPS),^[16]^ European Organization for the Research and Treatment of Cancer QLQ-C30 (EORTCQLQ-C30), Functional Assessment of Cancer Therapy-Colorectal (FACT-C), etc.
4. Adverse effects, focused on incidence of grade 2 or greater myelosuppression (hemoglobin, leukocyte and thrombocyte decreasing) and gastrointestinal adverse reaction (constipation, diarrhea, nausea, and vomiting) measured based on Standard Classification of WHO^[17]^ or National Cancer Institute Common Terminology Criteria for Adverse Events (NCI-CTCAE).

### 2.4 Study selection

**Figure 1:**
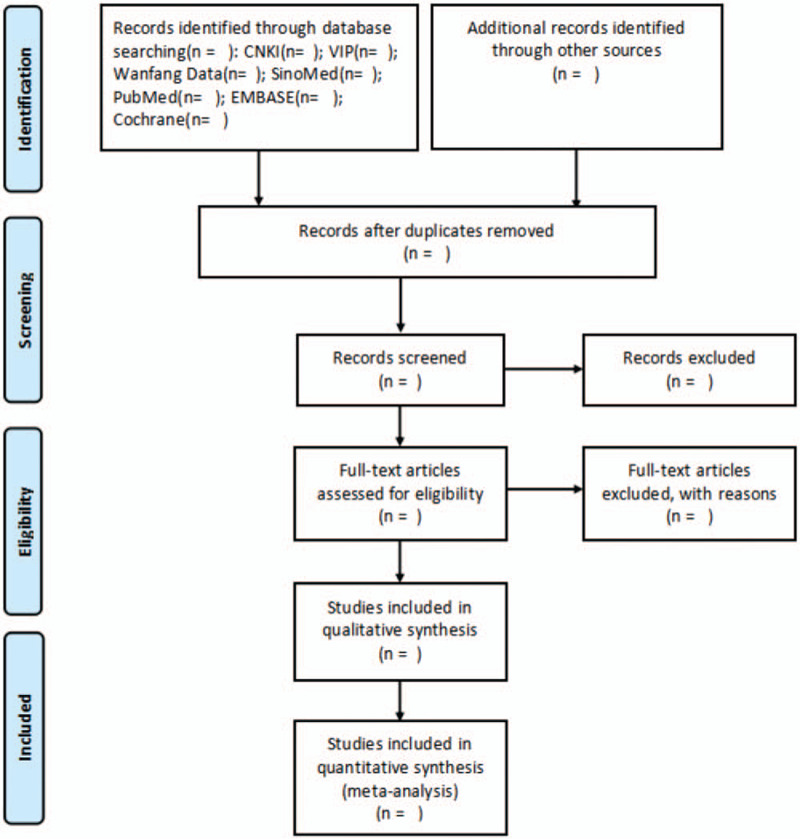
PRISMA flow diagram of study selection.

Search the database according to the established search strategies, and delete duplicates, the searched documents will be screened by reading the titles and abstracts on the basis of the inclusion and exclusion criteria, so that they can be included for the first time. The language is limited to English or Chinese. Then, the full-text will be read for further elimination. Use NOTEEXPRESS (version 3.0) to manage articles. The process of the study selection will be conducted by 2 authors independently. If there are any disagreements between the 2 authors, they will be decided by corresponding author. The procedures of study selection will be summarized through the PRISMA flow diagram (Fig. 1).

### 2.5 Data extraction

Two reviewers will complete the data extraction independently. The following items will be extracted: general information including author, year, publication, sample size, detailed information of participants, intervention measures of the treatment group and control group, outcomes, adverse events and possible conflicts of interest. The disagreements between the 2 reviewers will be settled by the third reviewer.

### 2.6 Quality assessment

Two evaluators will independently assess the methodological quality of the included articles by “Risk of Bias Assessment Tool” of the Cochrane Handbook for Randomized Controlled Trials.^[18]^ The risk of bias will be evaluated in 7 items including random sequence generation, allocation concealment, blinding of participants and personnel, incomplete outcome data, selective reporting, and other sources of bias, and finally evaluated as “low risk,” “unclear risk,” or “high risk.”^[19]^ Any differences will be decided by the third evaluator.

### 2.7 Data synthesis and analysis

#### 2.7.1 Data synthesis

Statistical analysis will be conducted by Revman 5.3 software. The heterogeneity will judged based on the *P* value and the *I*^2^ value. If the studies have non-significant heterogeneity (*P* > .1, *I*^2^ < 50%), we will use a fixed effects model. If there is great heterogeneity within the studies (*P* < .1, *I*^2^ > 50%), we will use the random effects model. If the data quantitative synthesis is not possible, we will analyze the available data qualitatively.

#### 2.7.2 Subgroup analysis and sensitivity analysis

We will perform a subgroup analysis if the collected data is sufficient, the following variables will be taken into account: the type of CHIs, the treating principle of CHIs based on TCM theories, the duration of CHI treatments, patients characteristics, etc. Finally, evaluation of the methodological heterogeneity will consider the risk of bias of the studies included. We will perform sensitivity analysis to determine the robustness of results.^[20]^

#### 2.7.3 Assessment of reporting biases

We will use a funnel-plot to assess publication bias if there are more than 10 articles for the meta-analysis.^[21]^

## 3 Discussion

CHIs, as one of the main innovations in the modernization of TCM, not only provide new options for advanced CRC treatments, but also provide new supplements for the administration routes of TCM.^[22]^ Although it had been widely used to combined with chemotherapy in advanced CRC and had proved to be functioned in enhancing efficacy and reducing toxicity in some clinical trials, to our knowledge, there is no published systematic review evaluating the efficacy of this combination therapy, particularly in all types of CHIs. Therefore, we decided to conduct this systematic review and meta-analysis to evaluate the efficacy of CHIs in advanced CRC patients and include those published randomized trials using fluoropyrimidine and oxaliplatin-based chemotherapy in both trial and control groups because this therapy is the main component of the first-line treatment for advanced CRC,^[23]^ so as to provide valuable evidence to apply CHIs in advanced CRC.

This review may have potential limitations. First, those important and widely-used CHIs were mainly used in China, which may lead to an unavoidable regional bias. Second, a quantitative analysis may not be possible as the lack of data in some outcomes. Third, some studies may lack methodological description like the methods of randomization and lead to a poor methodological quality.^[24]^

## Author contributions

**Conceptualization:** Wei Hou.

**Data curation:** Ying Zhang, Tong Zhou, Shuaihang Hu.

**Investigation:** Peiyu Tian, Zheng Li.

**Methodology:** Tong Zhou, Shuaihang Hu.

**Resources:** Peiyu Tian, Yuxiao Li.

**Supervision:** Yuerong Gui, Jun Dong, Wei Hou.

**Writing – original draft:** Shuo Wang, Xueqian Wang.

**Writing – review & editing:** Shuo Wang, Xueqian Wang, Ying Zhang.
